# Histone acetylation dependent energy landscapes in tri-nucleosome revealed by residue-resolved molecular simulations

**DOI:** 10.1038/srep34441

**Published:** 2016-10-04

**Authors:** Le Chang, Shoji Takada

**Affiliations:** 1 Department of Biophysics, Graduate School of Science, Kyoto University, Kitashirakawa, Sakyo 606-8502, Kyoto Japan

## Abstract

Histone tail acetylation is a key epigenetic marker that tends to open chromatin folding and activate transcription. Despite intensive studies, precise roles of individual lysine acetylation in chromatin folding have only been poorly understood. Here, we revealed structural dynamics of tri-nucleosomes with several histone tail acetylation states and analyzed histone tail interactions with DNA by performing molecular simulations at an unprecedentedly high resolution. We found versatile acetylation-dependent landscapes of tri-nucleosome. The H4 and H2A tail acetylation reduced the contact between the first and third nucleosomes mediated by the histone tails. The H3 tail acetylation reduced its interaction with neighboring linker DNAs resulting in increase of the distance between consecutive nucleosomes. Notably, two copies of the same histone in a single nucleosome have markedly asymmetric interactions with DNAs, suggesting specific pattern of nucleosome docking albeit high inherent flexibility. Estimated transcription factor accessibility was significantly high for the H4 tail acetylated structures.

In eukaryote, transcription is largely regulated by modulation of chromatin folding. Among other regulators, the histone tail acetylation has been known to play major roles to open chromatin structures and enhance transcription factor binding leading to activate transcription^1^,^2^,^3^,^4^,^5^. Among the four types of core histones, H3, H4, H2A, and H2B, acetylation in H4 N-terminal tail has been suggested to have major impact on chromatin folding and on gene expression activation^6^,^7^,^8^. Thus, the acetylation in the H4 tail is often used as a marker to monitor the level of transcription^9^,^10^,^11^. Roles of acetylation in other histones were less characterized.

Physically, highly basic histone N-terminal tails attractively interact with DNA as well as acidic patches on the surface of neighboring histone cores^12^ contributing to chromatin compaction. Acetylation in the histone tails removes a positive charge in the lysine side-chain and thus weakens such electrostatic attractions resulting in opening chromatin. Therefore, effects of histone tail acetylation can primarily be understood by the histone tail interactions in the un-acetylated case. The un-acetylated H3 and H4 tails mediate inter-nucleosome interactions via DNA^13^,^14^. H4 tail could also the interact with H2A acidic patch^6^,^15^,^16^,^17^,^18^. However, the interaction between H4 tail and DNA detected in an open nucleosome array is diminished after condensation of arrays^19^. The H3 tail contributes to chromatin compaction by screening the electrostatic interaction in linker DNA^20^,^21^,^22^. H2A and H2B tails do not mediate significant inter-nucleosome interaction^21^,^22^,^23^. Biochemical assays as well as computational studies with a mesoscopic model suggested that the strength of histone tail mediated chromatin compaction is in order of H4, H3, H2A, and H2B^21^,^22^,^23^.

Albeit many these studies, much of detail in histone tail interactions and in the effects of its acetylation is not elucidated yet. For example, experimentally, one can examine the effect of acetylation of individual lysine residue, but the lysine residue in 2*n* copies of the same tail in an *n*-nucleosome array cannot be distinguished. On top, interactions are normally detected via cross-linking, which monitors the distance proximity, but not interaction strength or overall structure^12^,^19^. Moreover, chromatin fiber is highly dynamic so that DNA sequence is accessible even when chromatin is visibly condensed^24^,^25^. Such dynamic motion of chromatin fiber is in <10-nm scale and difficult to be directly observed in experiments^26^. Understanding these dynamic motions at high resolution would serve as basis for the putative 30-nm chromatin fiber^27^,^28^,^29^,^30^ and 3D chromosome structure modeling^31^,^32^.

Given these situations, as a complementary approach to experiments, molecular dynamic simulations provide means to quantitatively investigate time-dependent structural dynamics of chromatin folding. Notably, however, fully atomistic molecular dynamics (MD) simulations are too time consuming to capture flexible and wide range of the structure ensemble of such a large system: only independent histone tails have been well studied by atomistic MD^33^,^34^. Whereas, the mesoscopic computational modeling has been very successful^21^, directly giving overall structural information. But, the mesoscopic resolution does not capture individual residues in histone tails. A very recent study based on multiscale computations showed promising results of bridging mesoscopic modeling with a higher resolution simulation^35^. Along a similar line, the purpose of current work is to extend the computational modeling to a higher resolution that represents all the residues, based on a multiscale approach, and decipher detailed interactions of histone tails and effects of their acetylation.

We have been developing and applying generic coarse grained (CG) models for protein-DNA complexes where each amino acid in proteins is represented by one particle^36^,^37^,^38^ and each nucleotide in DNA is approximated by three particles, each for phosphate, sugar, and base^39^. Interactions therein were tuned partly based on atomistic force field and partly by experimental data. For globular domains of proteins, the structure-based modeling with the fluctuation-matching algorithm provides high accuracy in representing native-fluctuations^36^,^37^. For intrinsically disordered regions of proteins, we took the sequence-based statistical potential, which was shown to give reasonably accurate representation^40^,^41^,^42^. Employing Langevin dynamics, one can obtain time-dependent structural information up to residue-resolution. For the nucleosome system, the CG model was first applied to study partial and mechanical DNA unwrapping from mono-nucleosome^43^. Then, inter-nucleosome interactions in di-nucleosome were briefly investigated^44^. We also studied structural dynamics of tri-nucleosomes with a SAXS/MD hybrid method: The SAXS profile computed from simulation ensemble agreed very well with the experimentally measured SAXS profile at a certain condition. Then, the structural feature in the simulated ensemble was characterized in details (Takagi *et al*. under review).

In this work, with the same CG MD method, we study structural dynamics of tri-nucleosome with several different histone tail acetylation states and analyze histone tail interactions with DNA and histone cores. Motivated by the energy landscape theory of protein folding^45^, we revealed the acetylation-dependent energy landscape of tri-nucleosome. The landscape view clarified that histone tail acetylation in each of four histones has unique and distinct features. The H4 tail acetylation had the largest effect on the radius of gyration by reducing interaction between the first and the third nucleosomes. The H3 tail acetylation reduced the electrostatic shielding on the neighboring linker DNAs, resulting in increase of the distance between neighboring nucleosomes. The H2A tail showed somewhat similar but weaker effect to H4 tail. The H2B tail acetylation also reduced the contact between the first and the third nucleosomes through indirect effects. Two copies of the same histone tail type in a nucleosome have sharply different interactions to DNA. For example, the tail of the second copy of the H4, but not the first copy, in the first nucleosome participates in interactions to linker DNAs and other nucleosomal DNAs. Additionally, to account for its relevance to transcription factor accessibilities, we estimated accessible surface area of a model transcription factor for DNA binding.

## Results and Discussions

### Computational Modeling of tri-nucleosomes with histone-tail acetylation

**Figure 1:**
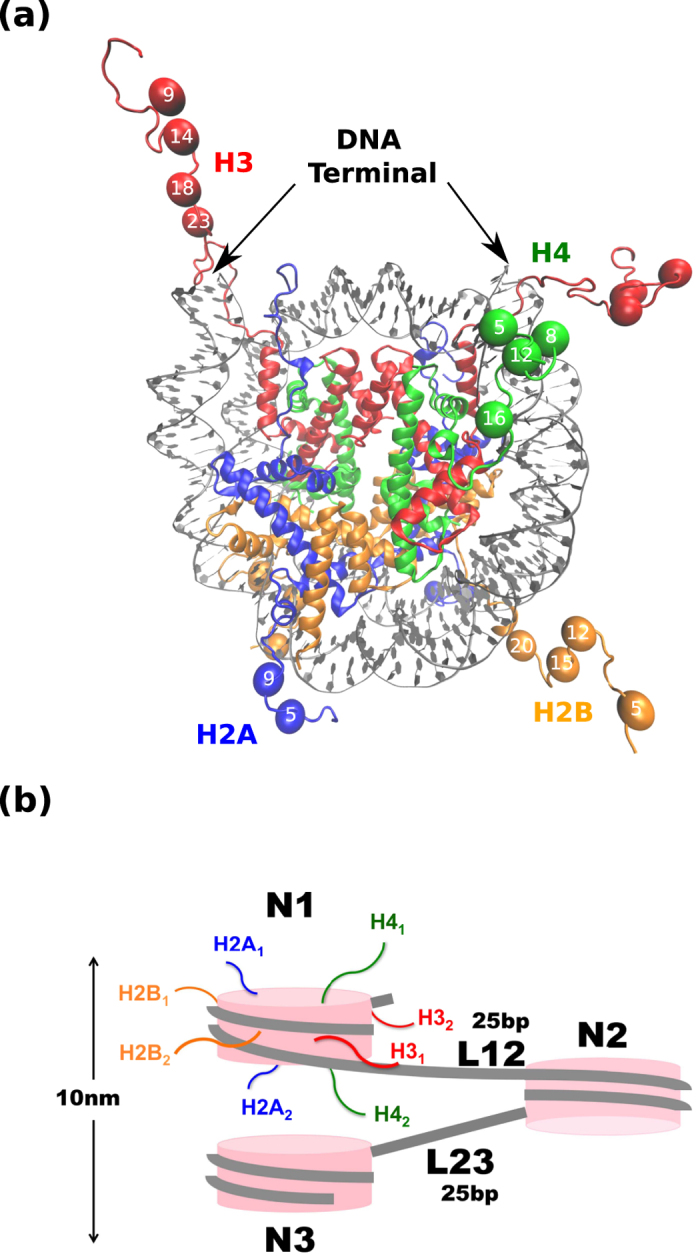
Mono- and tri-nucleosomes. (**a**) X-ray crystallographic model of mono- nucleosome (PDB code 1KX5). Colors indicate different type of molecules: histone H3 is in red, histone H4 is in green, histone H2A is in blue, histone H2B is in orange, and DNA is in gray. Spheres represent lysine residues in N-terminal tails that can be acetylated and numbers in the spheres indicate residue numbers. Structural image is generated by VMD. (**b**) Cartoon image of tri-nucleosome. We call the first, the second, and the third nucleosomes as N1, N2, and N3, while the first and the second linker DNAs are called L12 and L23, respectively. Two copies of each histone are labeled by the subscript 1 and 2.

The simulations system, tri-nucleosomes, contains three nucleosomes connected by two linker DNAs. Each nucleosome, of which structure is depicted in Fig. 1a, contains two each of four core histones, H2A (blue in Fig. 1a), H2B (orange), H3 (red), and H4 (green), which wraps 147 bp DNA (grey). The three nucleosomes are termed, in order, as N1, N2, and N3 (Fig. 1b). The N1, N2, and N3 nucleosomes are connected by the two linker DNAs of 25 bp long (Fig. 1b), which are termed as L12 (the linker between N1 and N2) and L23 (the linker between N2 and N3). We investigate effects of acetylation in N-terminal tails of histones. Specifically, we set a well-established set of lysine residues, K5 and K9 in H2A, K5, K12, K15, and K20 in H2B, K9, K14, K18, and K23 in H3, and K5, K8, K12, and K16 in H4 as possible sites for acetylation (indicated in Fig. 1a).

To make accurate and efficient conformational sampling possible, we employ a coarse-grained (CG) representation of tri-nucleosomes. Specifically, each amino acid in histones is represented by a single CG particle placed at Cα atom, while each nucleotide in DNA is represented by three CG particles: each representing phosphate group, sugar, and base. Using a structure-based CG model called AICG2+ for protein, histone cores largely keep their native structures throughout the simulations, while histone tails are treated as flexible chains^37^,^38^. For DNA, we used 3SPN.1 model, which prefers B-type double stranded form with a certain bending rigidity^39^. Within each nucleosome, histone-DNA interaction is based on the crystal structure so that the nucleosome core is largely maintained. For distant pairs of particles, steric repulsions and Coulombic interactions are applied with a dielectric constant of 78, where charges are placed at arginine (+1), aspartic acid (−1), glutamic acid (−1) and lysine (+1) in proteins as well as the phosphate group (−1) in DNA. For an acetylated lysine residue, we turned its charge to zero. Time propagation of tri-nucleosomes was realized by a standard Langevin dynamics with random stochastic force. All the simulations were conducted with the in-house developing software, CafeMol^46^.

We investigate structural dynamics of 6 sets of tri-nucleosomes with different acetylation states. The first set is the un-acetylated tri-nucleosome, which is precisely the same setup as we used in the hybrid SAXS/MD method to model the structural ensemble of tri-nucleosome (Takagi *et al*. under review). In that work, a certain simulation condition was identified that provides structural ensembles consistent with the solution X-ray scattering data. We used exactly the same simulation protocol here. This un-acetylated tri-nucleosome system serves as a control. For the next four setups, one of four histone tails in the tri-nucleosome was acetylated. In the case of H3 acetylation, for example, we turned off all the charges in K9, K14, K18, and K23 in 6 copies of H3. In the last setup, all four histone tails were acetylated, which is expected to give upper bound of the effects of acetylation.

### Simulations reveal distinct folding of tri-nucleosomes

**Figure 2:**
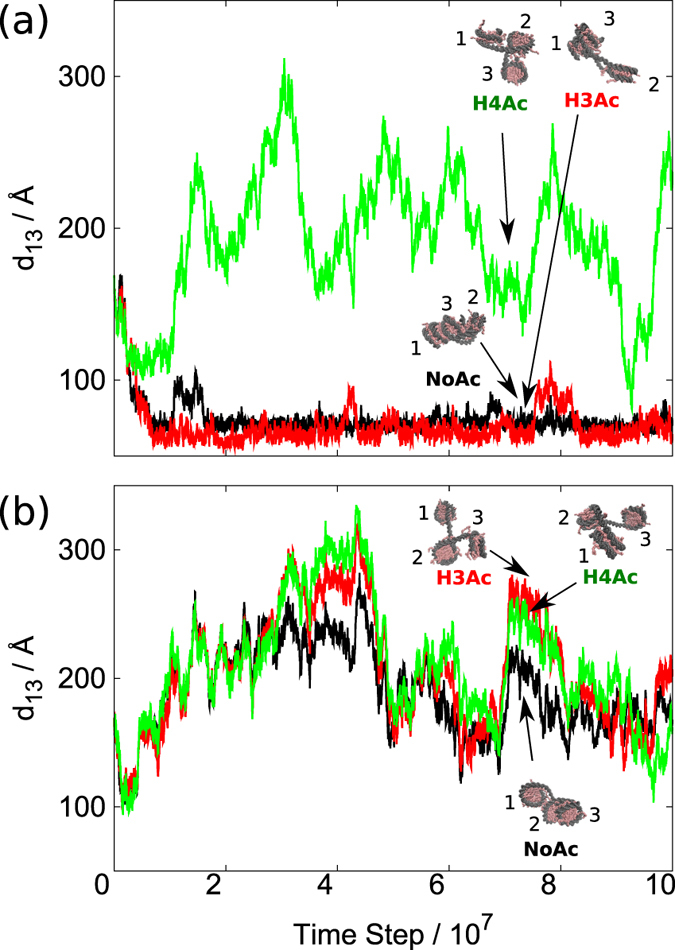
Representative time courses of the distance *d*_13_ between the first and the third nucleosomes in tri-nucleosomes simulations. (**a**,**b**) represent two representative trajectories with different stochastic forces. Un-acetylated (labeled as NoAc), H3-acetylated (H3Ac), and H4-acetylated (H4Ac) cases are drawn in black, red, and green, respectively. Representative snapshots are also depicted. Structural images are generated by VMD.

For each of 6 tri-nucleosome systems, starting from an extended conformation we performed CGMD simulations 10 times, each containing 10^8^ MD time steps (this can roughly be mapped to ~0.1 ms^44^). We illustrate folding of tri-nucleosomes in two different realizations of stochastic forces in Fig. 2a,b, where the distance *d*_13_ between the centers of nucleosomes N1 and N3 is plotted with the MD time step. The resulting trajectories exhibited rather diverse folding, depending of random stochastic forces. For the un-acetylated tri-nucleosome (black trajectories), the trajectory 1 (Fig. 2a) showed rather quick decrease in *d*_13_ to ~60–70 Å within 1 × 10^7^ MD time steps. This corresponds to docking of nucleosomes N1 and N3. Once realized, this docked state was maintained in the subsequent time. On the other hand, the trajectory 2 (Fig. 2b) led to a more open conformation with large fluctuation in *d*_13_ between 100 Å and 300 Å. Typically, in this open form, the two linker DNAs, L12 and L23 crossed each other, which is a characteristics found previously (Takagi *et al*. under review).

We next show two trajectories for H3-acetyalted tri-nucleosome (red in Fig. 2a,b) with the same realization of random stochastic forces as those in un-acetylated one. We find in Fig. 2 that H3-acetyaltion has little effect on the distance *d*_13_; the trajectory 1 resulted in the N1-N3 docking, while the trajectory 2 stayed in open conformations. Both trajectories are quite similar to those in the un-acetylated case.

We then plot two trajectories for H4-acetylated tri-nucleosome (green in Fig. 2a,b), which shows clear differences from the un-acetylated case. In the trajectory 1, we did not see docking between nucleosomes N1 and N3 leaving it to open-conformations, which are markedly different from the trajectory 1 of the un-acetylated and H3-aetylated cases. In the trajectory 2 of the H4-acetylated case, we find essentially the same time course as the un-acetylated and H3-acetylated cases. These two trajectories imply that the H4 acetylation destabilizes N1-N3 nucleosome docking, while open conformations are not very much affected by the same acetylation.

### Structural distributions

**Figure 3:**
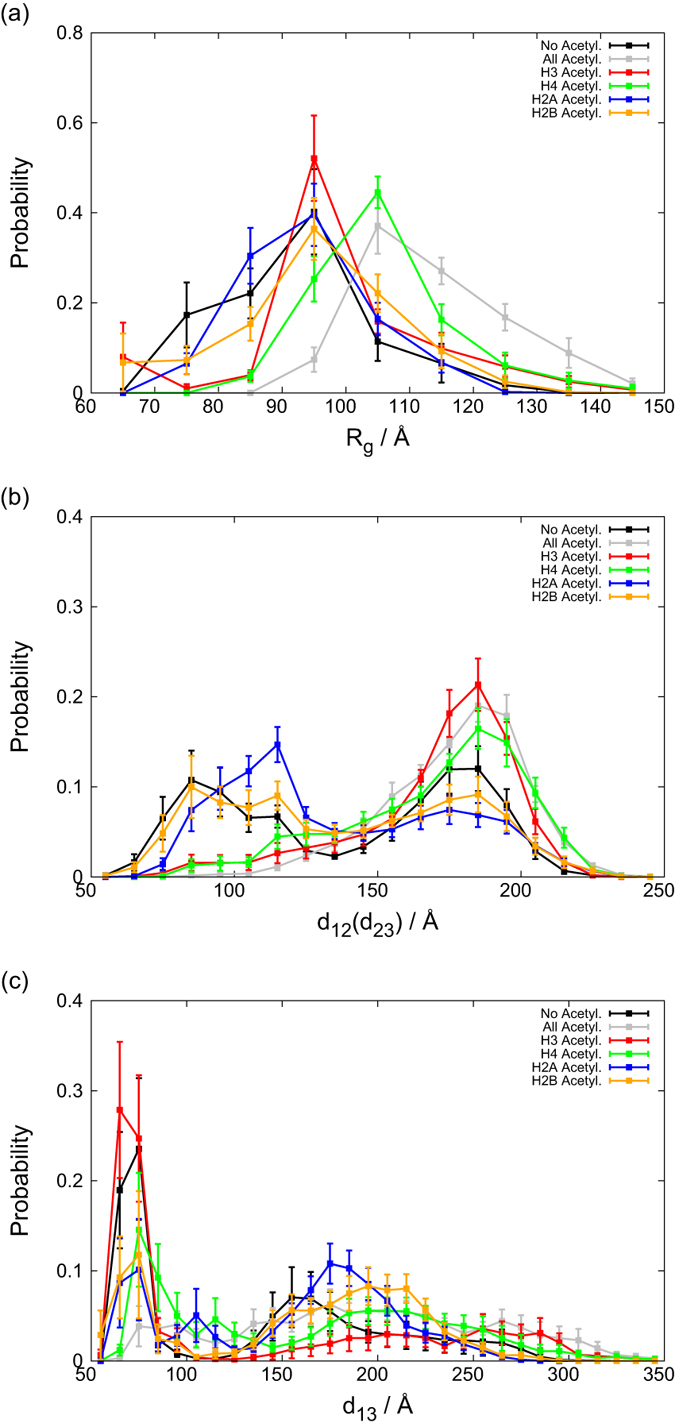
The simulated distributions of geometric parameters. (**a**) The radius of gyration, R_g_. (**b**) The distance between the centers of mass of neighboring nucleosomes. (**c**) The distance between the centers of mass of the first and third nucleosomes. Colors are used to distinguish different acetylation states as indicated in the figures.

For more quantitative analysis of structural feature of tri-nucleosomes, using the second half (which are used in all the following analyses) of the time courses of 10 trajectories in each of 6 setups, we obtained several probability distributions. We show three probability distributions in Fig. 3.

Figure 3a shows the distribution of the radius of gyration *R*_g_. We find that the un-acetylated (black), H2A- (blue), H2B- (orange), and H3-acetylated (red) tri-nucleosomes all showed the largest peak at *R*_g_ ~ 97.5 Å, while the H4 acetylated case (green line) has the largest peak value of 102.5 Å somewhat larger than the former cases. The setup with all-histone tails acetylated exhibited the peak at even larger value 107.5 Å (gray line). Thus, in consistent with the above observation in *d*_13_ trajectory, of the four histone tails, the H4 tail acetylation seems to have the largest impact on the compact folding of tri-nucleosomes. We note also that the un-acetylated case (black line) showed the secondary peak appears at 77.5 Å, which corresponds to highly docked states. Such a bi-modal distribution implies at least two distinct folding states present in un-acetylated case. It should be noted that the distribution for un-acetylated tri-nucleosome is the same as that we reported recently where these distribution was shown to give the simulated SAXS profile highly matching with the experimental SAXS profile (Takagi *et al*. under review).

We next look into the distributions of the distance *d*_12_ or *d*_23_ between the centers of neighboring nucleosomes (Due to the symmetry, the *d*_12_ and *d*_23_ are statistically indistinguishable so that we merged them in Fig. 3b. Hereafter, we denote it as *d*_12_ for simplicity). Interestingly, un-acetylated (black), H2A- (blue), and H2B-acetylated (orange) tri-nucleosomes showed clear bi-modal distributions. The un-acetylated case has its peaks at 85 Å (termed as the “tight state”) and 185 Å (the “loose state”), while the two peaks shifted to 115 Å (tight state) and 175 Å (loose state) for the H2A-acetylated case. In the tight state, the neighboring nucleosomes are in direct contact. On top, the peak for the tight state in the H2A acetylated case is somewhat higher than the un-acetylated case. Such changes mean that H2A acetylation could make neighboring nucleosomes in contact with slightly higher probability. In contrast, the H3- (red) and H4-acetylated (green) cases together with all (gray line) acetylated case possess only single peak at 185 ± 5Å corresponding to the loose state. Thus, both H3 and H4-acetylations destabilize the contact of neighboring nucleosomes.

Third, we plot the distributions of the distance *d*_13_ in Fig. 3c, where there are two major peaks in the distribution of all cases except the case of all-tails acetylated. The first peak at 70–80 Å corresponds to the N1-N3 docked state (termed as the “closed state”), while the second broad peak at larger distances corresponds to the “open state”. The probabilities for the closed state are relatively high for un-acetylated (black) and the H3 acetylated cases (red line). On the other hand, H2A, H2B, and H4 acetylation make compaction less probable. With all tails acetylated (gray), a peak corresponding to the closed state disappeared. In the open states, the distance *d*_13_ is inherently broadly distributed.

**Figure 4:**
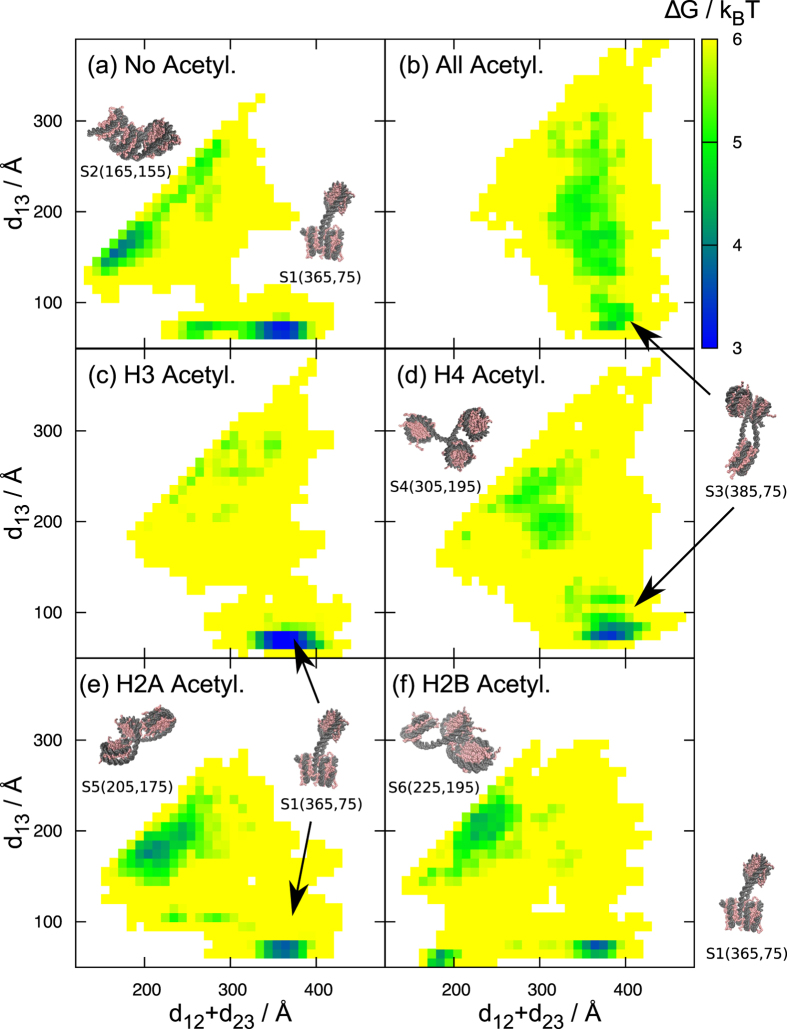
The free energy landscapes of six different acetylation states: Un-acetylated (**a**), all acetylated (**b**), H3 acetylated (**c**), H4 acetylated (**d**), H2A acetylated (**e**) and H2B acetylated (**f**) cases. Six representative structure images are drawn. The label below each structural image indicates the corresponding structure id (from S1 to S6) and numbers in brackets are d_12_ + d_23_ and d_13_ distances. Structural images are generated by VMD.

To better understand versatile conformational space, we then plot the free energy (Δ*G* = −*k*_B_*T* ln *P*, where *P* indicates the probability) landscape in the two-dimension (*d*_12_ + *d*_23_, *d*_13_) for all the 6 setups in Fig. 4. Representative structures located at peaks are also depicted with the symbols S*n* (*n* = 1, 2… and 6). Roughly, the distance *d*_13_ indicates tri-nucleosomes are either open (*d*_13_ > 100 Å, S2, S4, S5 and S6 are the examples) or closed (*d*_13_ < 100 Å, S1 and S3 are the examples) form, while *d*_12_ + *d*_23_ monitors whether neighboring nucleosomes are either loose (*d*_12_ + *d*_23_ > 200 Å) or tight (*d*_12_ + *d*_23_ < 200 Å). S1 is a tight and closed structure with N1 and N3 docking, which are found in the un-acetylated, H3-, H2A- and H2B- acetylated setups. S2 is a tight and open structure appeared only in the un-acetylated case.

Interestingly, looking the overall patterns of 6 setups in Fig. 4, we notice that none of the pairs is identical in this two-dimensional distribution, highlighting sensitivity and complexity in folding of histone-acetylated chromatin. In the plots, we find several densely located states, in particular in the un-acetylated (Fig. 4a), the H2A-acetylated (Fig. 4e), and the H2B-aectylated (Fig. 4f) setups. Each of these states tends to be closed or tight with inter-nucleosome contacts. The other three setups contain broader distributions. The H3-acetylated tri-nucleosome has few contacts in neighboring nucleosomes, as mentioned above. The H4-acetylated setup has a broad distribution with *d*_13_ > 100 Å. S4 is a representative dynamic open structure with crossed linker DNA’s.

As mentioned above, during simulations, once the tri-nucleosome falls into the closed state with the distance *d*_13_ < 70–80 Å, it hardly returns to the open state. This implies a high free-energy barrier and possible statistical bias in the probability distributions and the free energy landscapes. To test the convergence of sampling, we performed two additional calculations for the un-acetylated case, which contains the strongest electrostatic interactions and the highest free energy barrier in the 6 setups. First, we performed additional 15 independent 10^8^ step MD runs. With totally 25 trajectories, the probability distributions shown in Fig. S6 in Supplementary information (SI) are not significantly different from those in Fig. 3. Second, we conducted the umbrella sampling to compare the free energy difference between the closed state and the extended state (details are in Methods); the ratio *b* = P(70 Å < *d*_13_  < 80 Å)/P(200 Å < *d*_13_ < 210 Å). Based on the first 10 MD runs, we obtain, *b* = 12 ± 8. Based on the total 25 MD trajectories, *b* = 11 ± 5. By the umbrella sampling and the subsequent calculations of potential of mean force we obtain *b* = 12.7 (Figs S7 and S8 in SI). These together support the convergence of sampling with 10 MD trajectories within the estimated statistical errors.

### Electrostatic interaction analysis

**Figure 5:**
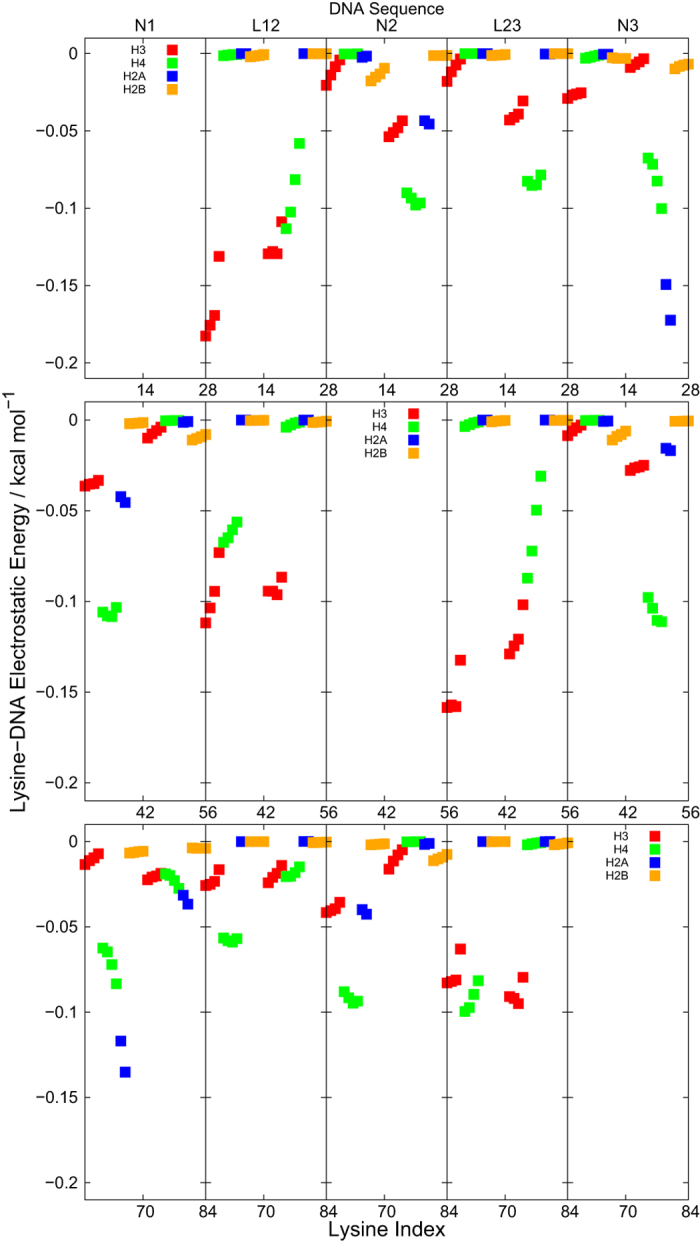
The electrostatic interactions energies between lysine residues that can be acetylated and 5 DNA fragments for un-acetylated tri-nucleosome. Lysine index are divided into three nucleosomes, each of which contains 28 lysine residues. The 28 lysine residues are further divided into two halves; each copy of H3, H4, H2A and H2B histones contain 14 lysine residues. Colors are used to distinguish lysine in different type of histones: H3 is in red, H4 is in green, H2A is in blue and H2B is in orange.

To obtain more microscopic insights on the roles of histone tails in tri-nucleosome folding, we next analyze site-specific interactions between histone tails and DNAs. For the un-acetylated tri-nucleosome, we calculated and plotted in Fig. 5 the average electrostatic interaction energies between each lysine that can be acetylated and each of 5 DNA fragments (N1, L12, N2, L23, and N3 defined in Fig. 1b). All interaction energies were obtained by averaging structural snapshots in the second half of time for 10 trajectories. Such energy value should be attractive and thus are negative since lysine has positive charge and DNA is negatively charged. Large absolute values of interactions suggest their importance and thus large effects in the acetylation of the lysine.

We first address interactions between the histone tails of the first nucleosome N1 with DNA. As mentioned above, one nucleosome contains 28 lysine residues that can be acetylated (4 in each of H3, H4, and H2B and 2 in each H2A). In the first row of Fig. 5, we plot interactions between each of 28 lysine residues (indexed from 1 to 28, differently colored by histone types) in the histone tail of N1 and 4 segments of DNA (L12, N2, L23, and N3). The intra-nucleosome interactions between lysine in N1 with the DNA fragment in N1 are much stronger and thus plotted separately (Fig. S1 in SI). In the first row of Fig. 5, we find the strongest interactions for lysine in the histone tail of the first H3 copy with the linker DNA L12. Of the four lysine residues in H3, those closer to N-terminus have stronger interactions. The H3 tail is located around the entrance/exit of each nucleosome, and thus this strong interaction stabilizes the nucleosome N1 by stapling the terminus of nucleosomal DNA^43^. Consistently with this view, interactions are asymmetric between two copies of H3; only the histone tail of the first H3, but not the second, has strong interaction (See Fig. 1b for the cartoon). Such finding could explain H3-acetylation enlarged the distance between neighbor nucleosomes (Fig. 3b). Next, we find markedly strong interactions for lysine in the second, but not the first, H4 molecule of N1 with all the four DNA segments; especially, attractions to the N2 and N3 nucleosomes must have contributed to tightening and closing of the (un-acetylated) tri-nucleosome, respectively. These findings could explain that H4-acetylated tri-nucleosome enlarged the distance between N1 and N2, as well as N1 and N3 (Fig. 3b,c). When two nucleosomes are docked each other at their planar surface (such as the N1 and N3 nucleosomes in the S1 structure), we often find the H4 tails are sandwiched by the two nucleosomes (Fig. 1b). Another noticeable interaction is the H2A tail between N1 and N3 nucleosome, which also contributes to closing of tri-nucleosome (Fig. 1b).

Electrostatic interactions of lysine in N2 (indexed from 29 to 56) with DNA fragments are plotted in the second row. General tendency found here is essentially the same as that in the first row. The asymmetry in the interactions is more outstanding. The histone tail of the first H4 interacts with L12 and N1, while the second H4 interacts with L23 and N3. The third row that represents interactions in the lysine in N3 (index from 57 to 84) has essentially the same information as the first row.

Effects of H2B acetylation are more subtle. In experiment, acetylation of H4 and H2B tails have largest effect to open nucleosome array^47^. However, H2B tail mediated inter-nucleosome interactions could not be detected and H2B tail-DNA interaction could not be weakened by acetylation^48^. In Fig. 5, we do not see any noticeable interactions of H2B tails, while H2B acetylation reduced closed conformation significantly (Fig. 3c) just as experimental results mentioned above. It should be noted that H2B acetylation weakened the interaction between histone tails and the DNA of the same nucleosome (Fig. S1 in SI), which enhances partial unwrapping of nucleosomes^43^,^49^. Indirectly, this might affect opening of tri-nucleosome.

In addition to lysine-DNA interaction, interaction between histone tail lysine and histone core acidic residue is also analyzed since the acidic patch in the histone core surface has been suggested as an important site. Results are qualitatively similar to lysine-DNA interaction: H4 tail mediates the strongest inter-nucleosome interaction (Fig. S2 in SI). However, the lysine-acidic patch interaction is weaker than lysine-DNA interaction due to smaller number of negatively charged residues.

### Conformational change in histone tails

**Figure 6:**
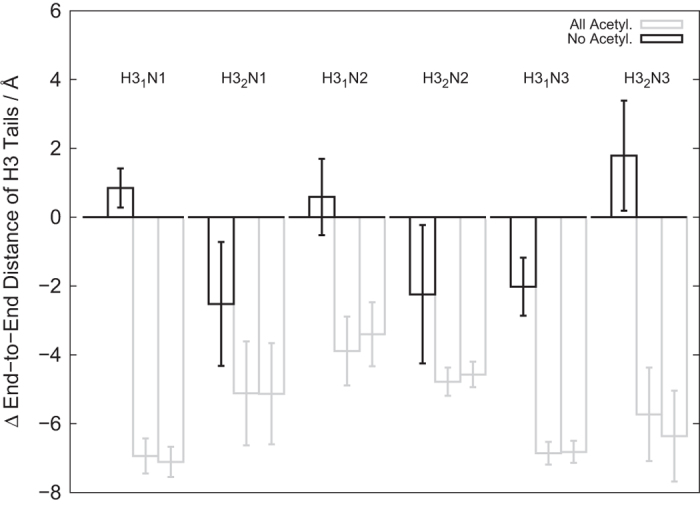
The average end-to-end distances of six individual H3 tails, relative to that for the closed state (*d*_13_ < 10 nm) of the un-acetylated case. The H3 tail is designated as H3_n_N_m_, where n is either 1 or 2 distinguishing two chains and m is 1, 2, or 3 indicating the nucleosome number. For each H3 tail, four data are shown; from left, the closed state of the un-acetylated case (for which the data are always zero by design), the open state of the un-acetylated case, the closed state of the all-acetylated case, and the open state of the all-acetylated case.

A recent study of chromatin dynamics with a multiscale protocol revealed that the H3 tails are extended in a compact chromatin while it is more folded in an open chromatin^35^. In that work, the flexibility of histone tails was modeled based on atomistic simulations of the tails^33^, which was put into mesoscopic modeling. In our work, we use one-bead-per-residue resolution throughout the work. Given the difference in modeling methods, we ask if we obtain the same tendency as the previous work in the histone tail folding coupled with chromatin opening. To do this, using 10 MD trajectories, we calculated the average end-to-end distance for each histone tail both in the closed structure ensemble (defined as *d*_13_ < 10 nm) and in the open structure ensemble (defined as *d*_13_ > 10 nm). The histone tails are defined as the N-terminal 38, 26, 23, and 14 residues in H3, H4, H2B, and H2A, respectively, following the previous works^33^,^35^. The average end-to-end distance for each histone tail is listed in Tables S1 and S2 in SI and is plotted for the H3 tail in Fig. 6.

In Fig. 6, for individual H3 tails, changes in the average end-to-end distances (relative to that in the compact structure ensemble) are plotted. We see that the relative end-to-end distance in the open structure ensemble is negative for some H3 tails (the second copy of H3 in N1, the second copy of H3 in N2, and the first copy of H3 in N3), indicating that these H3 tails are more folded in the open chromatin. The behavior of these H3 tails is consistent with the previous work. By visual inspection, we found that these H3 tails are free from the flanking linker DNA and thus can interact with other nucleosomes or distant linker DNA (See the second copy of the H3 tail in N1 in Fig. 1b as illustration). Other H3 tails, for which no significant changes were observed in Fig. 6, are located along the flanking linker DNAs (See the first copy of H3 tail in N1 in Fig. 1b as illustration). These electrostatic interactions between the H3 tails and the flanking linker DNA are so strong that the conformations of these H3 tails are not affected by the higher-order chromatin folding. Interestingly, each linker DNA is occupied by only one H3 tail (Note that this may be true only for relatively short nucleosome repeat lengths). For example, the L12 linker DNA is occupied by the first copy of H3 tail in N1. Thus, the second copy of H3 tail in N2 cannot interact strongly with L12, making it possible to interact with other nucleosomes. Such explanation is well consistent with the lysine-DNA electrostatic interaction energies shown in Fig. 5.

Such behavior disappears when histone tails are acetylated (gray boxes in Fig. 6), indicating the compaction of H3 tails in open structures is related to electrostatic interactions.

### Accessibility of transcription factors

Finally, we briefly address biological significance of acetylation-dependent tri-nucleosome structures. We try to evaluate how accessibility of a transcription factor is altered by different folding structures. Here, we model TF as a sphere with the radius of 30 Å and quantify its accessibility to DNA by the transcription factor accessible surface area (TFASA) (Fig. S3 in SI), which we calculate in several different conformations (Sn, with n = 1~6). For each state Sn, the TFASA was obtained by the average of many structures corresponding to the same state in the 2D structural free energy landscape (Fig. 4). Detailed method for computing the TFASA is described in the section “TFASA” and SI. Results with different radii of TF are given in Fig. S5 in SI.

**Figure 7:**
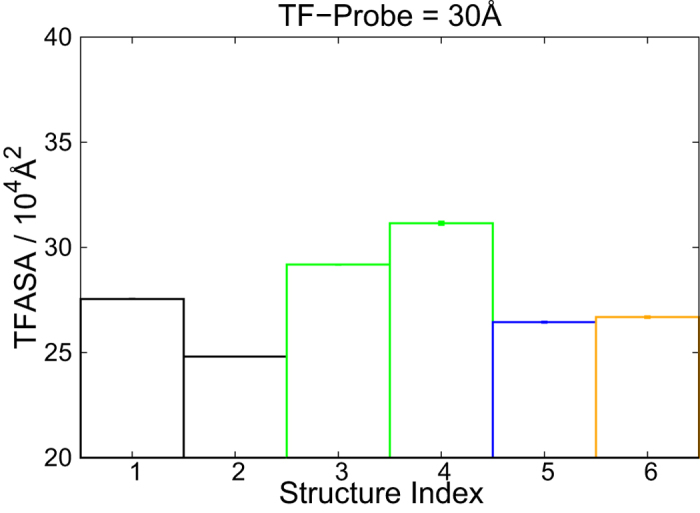
The transcription factor accessible surface area (TFASA) for 6 representative structures (Sn, with n = 1, 2…, 6). Each value of TFASA (with error bar) is the average of many corresponding structures in energy landscape (Fig. 4). Colors used to represent the dominant acetylation state of corresponding structure: no acetylation is in black, H4 acetylation is in green, H2A acetylation is in blue and H2B acetylation is in orange.

The TFASA for 6 representative structures, S1 to S6, are presented in Fig. 7. Clearly, structures appeared in the H4-acetylated case (S3 and S4, green) have larger TFASA than other structures although the difference is modest. It is interesting that, even though S3 is the closed conformation, S3 possess a relatively large TFASA. Whereas, even with the open conformation, structures corresponding to un-acetylated (S2, black box), H2A acetylated (S5, blue box) and H2B- acetylated (S6, orange box) systems have smaller TFASAs. Such finding indicates whether there is sufficient space for TF binding is not simply decided by the opening/closing of tri-nucleosome. Without sufficient structure loosening, TF binding is relative difficult even for open states (S2, S5 and S6 in Fig. 7). Such finding is consistent with an experimental observation that a histone-tail acetylation does not alter compaction but activate gene expression^50^. Other studies revealed that transcription factors could bind to structurally inaccessible region of chromatin^51^,^52^, which implies there is space for TF binding even in a compact chromatin structure. However, such space will be small. When we used too large radius of TF (over 60 Å, Fig. S5 in SI), the difference among different structures was reduced since there is no space for TF binding. Therefore, the sensitivity to distinguish TFASA among different folding structures depends, to some extent, on the size of TF probe.

**Figure 8:**
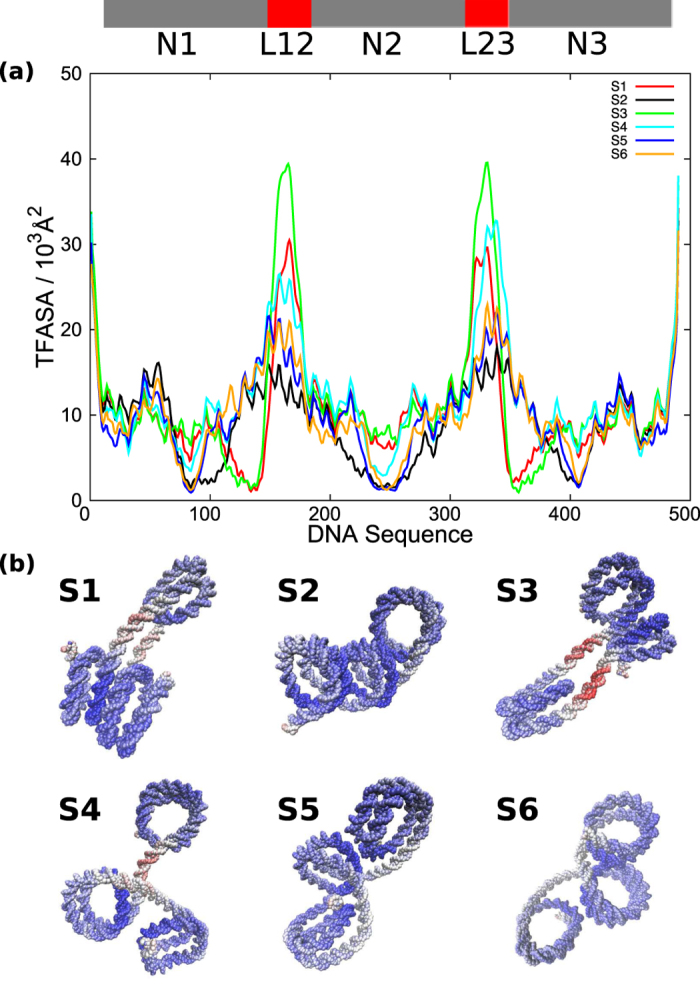
The local transcription factor accessible surface area (TFASA) for 6 representative structures (Sn, with n = 1, 2…, 6). (**a**) The local TFASA is plotted against the DNA sequence. (**b**) The TFASA is represented as colors on the structures. The red, white, and blue indicate large, medium, and small TFASAs, respectively. Structural images are generated by VMD.

TFASA computed from typical structures is sufficient to distinguish accessibility to entire DNA sequence, but is not enough to detect which part of DNA sequence can be accessed most. Therefore, the local TFASA for every base pair along DNA sequence are computed (Fig. 8). In Fig. 8a, the local TFASA along DNA sequence are shown. Colors are used to distinguish structure index from S1 to S6. Due to nucleosome occupancy (indicated by grey bars at the top), N1, N2, and N3 DNA fragments reveal lower values of TFASA, while linker regions (L12 and L23) give higher values of TFASA, consistent to recent experimental results by ChIP-seq or DNase-seq technique to identify nucleosome position^53^. Figure 8b gives TFASA values by structural image. Notably, while S1 and S3 apparently look similar in structure, S3 has markedly larger TFASA values (represented by red) in linker regions than those in S1. The linker DNA in S3 tends to be straighter and thus can be accessed by TFs more.

## Conclusion

We investigated histone tail acetylation dependence of the free energy landscape of tri-nucleosome using molecular simulations that has the residue-level resolution. We found that the tail acetylation in each histone alters the energy landscape in distinct manner. Of the four histones, the H4 tail acetylation showed the largest change; the open and loose states became dominant. The H3 tail acetylation increased the distance between neighboring nucleosomes. In the analysis of un-acetylated histone tail interactions with DNA, we found that two copies of each histone tail in each nucleosome show markedly different interactions, suggesting specific pattern of nucleosome docking. As for the histone tail mediated inter-nucleosome interactions, we not only obtained results consistent to experimental data for the H3- and H4- tails, but also suggested some interactions for H2A- and H2B- tails, which are difficult to detect in experiments (A brief summary is in Table 1). We also showed that the change in tri-nucleosome structure is correlated with altered accessible surface area of generic transcription factors: The H4 acetylated system showed the largest accessibility.

## Methods

### Coarse-grained (CG) molecular dynamic (MD) simulations

In CG MD of this work, histone globular domains were mostly restrained to their crystal structures by AICG2+ potential^37^,^38^. Intrinsically disordered histone tails were modeled as flexible chains depending on local structural propensities^40^,^42^. The DNA was modeled with 3SPN.1 model, which biases double stranded DNA to the B-type form and can bend by interacting with histones^39^. The total energy function for CG MD consists of four components:
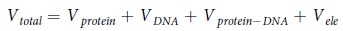


Detail information of energy functions are described in SI, The last term of electrostatic potential is in Debye-Hückel form. As for the salt concentration, we used 100 mM which reproduces structures compatible with the small-angle X-ray scattering (SAXS) experimental profiles (Takagi *et al*. under review).

Starting from X-ray crystallographic structure (PDB code 1KX5), three copies of nucleosome DNA (147bp) are connected by two 25 bp linker fragments, followed by energy minimization with AMBER^54^. This minimized structure was used as the initial configuration of CG MD for all trajectories. Time propagation in the CG MD was modeled by the standard Langevin dynamics. The single MD step can be mapped to ~1 ps^44^.

We estimated the statistical error in Fig. 3 by computing the probability distributions for each trajectory, from which we obtained the standard deviation. Assuming the independence of all the trajectories, we estimated the standard error. The same approach was used for the error bar in Fig. 6.

### Transcription Factor Accessible Surface Area (TFASA)

To compute TFASA, we split the 3D space into grids with the edge length *g*. On each grid point, we put a sphere TF probe with the radius *R*. The number *N* of points where the probe is “in contact” to DNA was enumerated (Fig. S3). The definition of “in contact” is by the distance between the probe and CG bead closest to the probe being in the range from *R* to *R* + 10 Å. The probe could not be placed at any overlapping point (distance to CG particle from 0 to R) because of the excluded volume. The TFASA was defined as *Ng*^3^/10 Å. The denominator 10 Å represents a thickness of the surface in computing area. Detail information such as the grid size and the probe radius is given in SI.

### Umbrella sampling simulations

To estimate the height of free energy barrier between the closed state (*d*_13_ < 10 nm) and the open state (*d*_13_ > 10 nm), the umbrella sampling is performed. From 50 Å to 250 Å of *d*_13,_ we set 101 equally spaced centers with the gap of 2 Å. With the spring potential restraint to each center, we performed 10^7^ step MD trajectories. The spring constant is 0.1 kcal mol^−1^ Å^−2^. The initial structure of umbrella sampling is the same as conventional MD. The first 2 × 10^6^ steps are discarded. To calculate the canonical-ensemble probability, we utilized the weighted histogram analysis method (WHAM)^55^.

We note that, using *d*_13_ alone as the reaction coordinate, we sampled the closed state in which the nucleosomes N1 and N3 dock each other and the extended state, but not the tight state where the adjacent nucleosomes dock each other. The sampled area in the umbrella sampling is plotted in Fig. S8, which clearly shows this simulation does not cover the tight state. Thus, we used the umbrella sampling simulation solely to compare the free energy of the closed state with that of the extended state, but not with the tight state.

## Additional Information

**How to cite this article**: Chang, L. and Takada, S. Histone acetylation dependent energy landscapes in tri-nucleosome revealed by residue-resolved molecular simulations. *Sci. Rep.* **6**, 34441; doi: 10.1038/srep34441 (2016).

## Supplementary Material

Supplementary Information

## Figures and Tables

**Table 1:** Summary of key roles of each histone tail obtained by experiments and by our results.

Name of Tail	Experimental tail-mediated nucleosome Interaction	Experimental effect of acetylation (ref. 47)	Our result
H3	Cross-linking of tail-DNA, H1 binding more effective than actylation (ref. 13)	DNA unwrapping	Interact with linker DNA
H4	Cross-linking of tail-DNA and H4 tail-H2A, H1 binding less effective than actylation (ref. 14)	Nucleosome Array Opening	Interact with other nucleosome DNA
H2A	Not Detected	Unclear	Interact with other nucleosome DNA with strength weaker than H4 tail
H2B	Not Detected	Nucleosome Array Opening	Interact with self nucleosome DNA
