# Supplementary material for: Histone acetylation dependent energy landscapes in tri-nucleosome revealed by residue-resolved molecular simulations
Source: Sci Rep. 2016 Oct 4;6:34441. doi: 10.1038/srep34441 (PMC5048180; doi:10.1038/srep34441)
Supplement: Supplementary Information [file srep34441-s1.doc]

**Histone acetylation dependent energy landscapes in tri-nucleosome revealed by residue-resolved molecular simulations**

**Le Chang^1^ and Shoji Takada^1*^**

^1^Department of Biophysics, Graduate School of Science, Kyoto University, Kitashirakawa, Sakyo, Kyoto Japan 606-8502

**Coarse-grained (CG) molecular dynamic (MD) simulations**

We have been developing and applying coarse-grained (CG) models for proteins and DNA where each amino acid in proteins is represented by a single CG particle placed at Cα atom and each nucleotide in DNA is represented by three CG particles: phosphate group, sugar, and base. Histone globular domains were mostly restrained to their crystal structures by AICG2 potential ^1,2^. Intrinsically disordered histone tails were modeled as flexible chains depending on local structural propensities^3,4^. The DNA was modeled with 3SPN.1 model, which biases dsDNA to the B-type double stranded DNA and can bend by interacting with histones ^5^.

The total energy function for CG MD consists of four components:

$$V_{total}=V_{protein}+V_{DNA}+V_{protein-DNA}+V_{ele}$$

For proteins, AICG2+ energy function is used ^1^.

Here, the first term restrains the lengths of virtual bonds between adjacent amino acids; the second term represents sequence dependent angle and dihedral-angle potentials described below. The third and the fourth terms are local potentials biased to the native structure. The fifth term is the attractive potential between pairs of amino acids that are in contact at the native structure. The last term is a simple short-ranged repulsion potential between pairs of amino acids not contacted in the native structure.

The second term for the protein potential, the flexible local potential, is a sum of the two contributions: the bond angle potential and the dihedral angle potential, both obtained via the Boltzmann inversion of probability distributions in a loop library extracted from the protein data bank ^3^.

To ensure the periodicity in φ, we used a truncated Fourier series

$$V_{dih}\left(\varphi\right)=\sum_{m}^{3} k_{m}\sin\left(m\varphi\right)+\sum_{n}^{3} k_{n}\cos\left(n\varphi\right)+C$$

Notably, for intrinsically disordered regions, there is no bias to the native structure and only the first, the second and the last terms in *V*_protein_. The intrinsically disordered regions are defined as the first 15 N-terminal residues of H2A, the first 33 N-terminal residues of H2B, the first 43 N-terminal residues of H3, and the first 23 N-terminal residues of H4, according to B-factors in the crystal structure (1KX5 from the protein data bank).

For DNA, 3SPN.1 model was used ^5^.

For protein-DNA interactions, the following energy function was used.

$$V_{protein-DNA}=\sum_{j>i}^{nat-contact} \varepsilon_{go,ij}^{prot-dna}\left[5\left(\frac{r_{ij0}}{r_{ij}}\right)^{12}-6\left(\frac{r_{ij0}}{r_{ij}}\right)^{10}\right]+\sum_{j>i}^{non-native} \varepsilon_{exv}^{prot-dna}\left(\frac{d^{prot-dna}}{r_{ij}}\right)^{12}$$

The energy function only considers the bias to the native structure and the excluded volume similar to the last two terms in the energy function of protein, but with different strength.

The electrostatic interaction is in Debye-Hückel form with the dielectric constant of 78. Charges are only placed at arginine (+1), asparagine (-1), glutamine (-1) and lysine (+1) in protein as well as the phosphate group (-1) in DNA. As for the salt concentration, we used 100 mM, which was shown to reproduce structures compatible with the small-angle X-ray scattering (SAXS) experimental profiles (Takagi et al, under review).

Particle movement follows the standard Langevin equation:

$$m_{i}\frac{d^{2}r_{i}}{dt^{2}}=-\frac{\partial V_{total}}{\partial r_{i}}-m_{i}\gamma_{i}\frac{dr_{i}}{dt}+m_{i}\xi_{i}$$

Here, $i$ is the particle ID, $\gamma$ is the friction coefficient, $\xi$is Gaussian white noise with the mean and variance:

$$\begin{matrix} \left\langle\xi_{i}(t)\right\rangle=0, \\ \left\langle\xi_{i}(t)\xi_{j}(t')\right\rangle=\frac{2\gamma_{i}k_{B}T}{m_{i}}\delta(t-t')\delta_{i,j} \end{matrix}$$

$k_{B}$ is the Boltzmann constant, and $T$ is temperature and set to 300K (room temperature). In all the CG MD simulations, we used the software CafeMol ^6^.

Starting from X-ray crystallographic structure (PDB code 1KX5), three copies of nucleosome DNA (147bp) were connected by two 25bp linker fragments, followed by energy minimization with AMBER ^7^. This minimized structure was used as the initial configuration of CG MD for all trajectories.

**Lysine-DNA Electrostatic Energy in Each Nucleosome**

In the main text, we described interaction energies of lysine residues in histone tails with DNA fragments that do not wrap to the same histone octamers since these “non-local” interactions are directly related to folding of nucleosome array. However, obviously, lysine residues in histone tails interact with the DNA fragment which wraps the corresponding histone cores. These “local”-interactions are, to some extent, obligatory and thus tend to be stronger than “non-local” interactions. Here, we describe local interactions (Fig. S1). In Fig. S1, we find that the strongest interaction is from H2B tails, which tend to stay around the gap region of two DNA sub-fragments (Fig.1b). Such interaction stabilizes the structure of wrapped nucleosomal DNA. Therefore, acetylation of H2B tails will cause nucleosome unwrapping ^8,9^. Such unwrapping might be related to total chromatin decompaction by H2B acetylation in this work which is also found in experimental studies ^10,11^.

**Lysine-Acidic Patch Electrostatic Energy**

In addition to lysine-DNA interaction, lysine residues in histone tails interact with the so-called acidic patch (negatively charged residues on the surface of histone octamer) contributing to the stability of chromatin fiber structure. To quantify the strengths of different histone tails interacting with acidic patch, electrostatic energies are computed between each lysine residue in histone tails and acidic patch (Fig. S2). In Fig. S2, we find that energies for each histone tail are similar to energies in lysine-DNA interaction: H4 tail has strong interaction with acidic patch in neighbor nucleosomes, since the H4 and H2A tails make strong interaction with acidic patch in far distant nucleosomes. On top, the H2B tail has strong interaction with acidic patch in the neighboring nucleosome. Although the energy is similar to lysine-DNA interaction, the strength, as a total, is much weaker since the number of negatively charged residues in acidic patch is much smaller that the number of negatively charged phosphate in DNA.

**Transcription Factor Accessible Surface Area (TFASA)**

To compute TFASA, we split the 3D space into grids with the edge length *g*. On each grid point, we put a sphere TF probe with the radius *R*. The number *N* of points where the probe is “in contact” to DNA was enumerated (Fig. S3). The definition of “in contact” is by the distance between the probe and CG bead closest to the probe being in the range from *R* to *R*+10Å. The probe could not be placed at any overlapping point (distance to CG particle from 0 to R) because of the excluded volume. The TFASA was defined as *Ng^3^*/10Å. The denominator 10Å represents a thickness of the surface in computing area.

By such scheme, the accuracy of TFASA depends on the grid size *g*, of which dependence was investigated in Fig. S4. In Fig. S4, the error due to the grid size converged reasonably rapidly as grid size decreases. The error was sufficiently small below the grid size of 3Å, which will be used for further TFASA calculations. Here, the error was computed by repeating TFASA calculation with 12 different rotation angles of the same molecular structure.

As for the radius of TF probe R, various values form 10Å to 60Å were tested (Fig. S5). In Fig. S5, we see that the structures S3 and S4 corresponding to the H4 tail acetylation have higher values of TFASA for all the tested values of R. The increase of the corresponding TFASA relative to S1 reaches maximum at the radius of TF probe R = 30Å, which was used for further TFASA calculations. Smaller or larger radius is not effective to detect TFASA difference among structures.

**Umbrella sampling simulations**

To estimate the height of free energy barrier between closed state (*d*_13_<10nm) and open state (*d*_13_>10nm), umbrella sampling is performed. We have 101 independent 10^7^ steps trajectories with spring potential at different centers of *d*_13_, from 50Å to 250Å with a gap of 2Å. The spring constant is 0.1 kcal mol^-1^ Å-^2^. The initial structure of umbrella sampling is the same as conventional MD. The first 2x10^6^ steps are not used to make equilibrium samplings. To calculate the reweighted probability, the weighted histogram analysis method (WHAM) ^12^ is used.

The potential of mean force (PMF) of different *d*_13_ is showed in Fig. S7, where the lowest value of PMF appears at 75Å, which is consistent to Fig. 3c and Fig. S6c. Besides, there is a small valley at 110Å, which is not observed in Fig. 3c but detected in Fig. S6c. However, such a small valley will affect the convergence of entire sampling. The problem is the free energy minimum at 165Å is not detected. Such problem is caused by the spring potential between the first and third nucleosome in umbrella sampling. Such constraint could easily disrupt the interaction between the first and third nucleosome and estimate the free energy barrier between closed and open state. In the meantime, such constraint makes the second nucleosome difficult to approach either first or third nucleosome (Fig. S8). Fig. S8 is a scattered plot of *d*_12_+*d*_23_ for different value of *d*_13_ in umbrella sampling, where *d*_12_+*d*_23_ < 180Å is not sampled. In that sense, canonical MD is more efficient.

**References**

1. Li, W., Terakawa, T., Wang, W. & Takada, S. Energy landscape and multiroute folding of topologically complex proteins adenylate kinase and 2ouf-knot. *Proc. Natl. Acad. Sci. U. S. A.* **109,** 17789–94 (2012).

2. Li, W., Wang, W. & Takada, S. Energy landscape views for interplays among folding, binding, and allostery of calmodulin domains. *Proc. Natl. Acad. Sci. U. S. A.* **111,** 10550–5 (2014).

3. Terakawa, T. & Takada, S. Multiscale ensemble modeling of intrinsically disordered proteins: P53 N-terminal domain. *Biophys. J.* **101,** 1450–1458 (2011).

4. Terakawa, T., Higo, J. & Takada, S. Multi-scale ensemble modeling of modular proteins with intrinsically disordered linker regions: Application to p53. *Biophys. J.* **107,** 721–729 (2014).

5. Sambriski, E. J., Schwartz, D. C. & De Pablo, J. J. A mesoscale model of DNA and its renaturation. *Biophys. J.* **96,** 1675–1690 (2009).

6. Kenzaki, H. *et al.* CafeMol: A coarse-grained biomolecular simulator for simulating proteins at work. *J. Chem. Theory Comput.* **7,** 1979–1989 (2011).

7. Pearlman, D. A. *et al.* AMBER, a package of computer-programs for applying molecular mechanics, normal-mode analysis, molecular dynamics and free-energy calculations to simulate the structural and energetic properties of molecules. *Comput. Phys. Commun.* **91,** 1–41 (1995).

8. Ettig, R., Kepper, N., Stehr, R., Wedemann, G. & Rippe, K. Dissecting DNA-histone interactions in the nucleosome by molecular dynamics simulations of DNA unwrapping. *Biophys. J.* **101,** 1999–2008 (2011).

9. Kenzaki, H. & Takada, S. Partial Unwrapping and Histone Tail Dynamics in Nucleosome Revealed by Coarse-Grained Molecular Simulations. *PLo Comp Biol* **11,** e1004443 (2015).

10. Wang, X. & Hayes, J. J. Site-specific binding affinities within the H2B tail domain indicate specific effects of lysine acetylation. *J. Biol. Chem.* **282,** 32867–32876 (2007).

11. Wang, X. & Hayes, J. J. Acetylation mimics within individual core histone tail domains indicate distinct roles in regulating the stability of higher-order chromatin structure. *Mol. Cell. Biol.* **28,** 227–36 (2008).

12. Kumar, S., Bouzida, D., Swendsen, R. H., K~llma, P. A. & Rosenbergl, J. M. The Weighted histogram analysis method for free-energy calculations on biomolecules. I. the method. *J. Comput Chem* **13,** 1011–1021 (1992).

**Figures**

**Figure S1**: Lysine-DNA electrostatic energies with within the same nucleosomes.

**Figure S2**: Lysine-acidic patch electrostatic energies.

**Figure S3**: Schematic view of TF-probe.

**Figure S4**: TFASA error with different grid size.

**Figure S5**: TFASA values with different size of probe radius.

**Figure S6**: Convergence of un-acetylated state between 10 trajectories and 25 trajectories.

**Figure S7**: Height of free energy (PMF) barrier between closed state (*d*_13_ = 75Å) and open state (*d*_13_ = 205Å).

**Figure S8**: Scattered plot of *d*_12_+*d*_23_ for different value of *d*_13_ in umbrella sampling.

**Table S1**: End-to-end distance of closed structures (*d*_13_<10nm) and open structures (*d*_13_>10nm) for all histone tails without acetylation.

| **Tail Name** | **End-to-End Distance (d13<10nm)** | **End-to-End Distance (d13>10nm)** |
| --- | --- | --- |
| H31N1 | 45.98±0.40 | 46.83±0.41 |
| H41N1 | 35.37±0.16 | 35.81±0.43 |
| H2A1N1 | 25.40±0.06 | 25.53±0.10 |
| H2B1N1 | 37.22±0.09 | 36.93±0.09 |
| H32N1 | 45.26±1.47 | 42.74±1.04 |
| H42N1 | 34.02±0.06 | 34.67±0.16 |
| H2A2N1 | 24.45±0.05 | 25.10±0.05 |
| H2B2N1 | 37.40±0.11 | 37.51±0.09 |
| H31N2 | 42.98±0.92 | 43.57±0.62 |
| H41N2 | 35.54±0.10 | 35.52±0.46 |
| H2A1N2 | 25.47±0.08 | 25.35±0.12 |
| H2B1N2 | 37.12±0.04 | 37.09±0.12 |
| H32N2 | 44.52±0.33 | 42.28±1.98 |
| H42N2 | 35.12±0.11 | 34.89±0.11 |
| H2A2N2 | 25.42±0.10 | 25.32±0.05 |
| H2B2N2 | 37.64±0.13 | 37.52±0.08 |
| H31N3 | 46.30±0.30 | 44.28±0.78 |
| H41N3 | 34.47±0.23 | 34.86±0.28 |
| H2A1N3 | 24.62±0.18 | 25.19±0.10 |
| H2B1N3 | 37.14±0.18 | 36.94±0.11 |
| H32N3 | 45.51±1.31 | 47.30±0.92 |
| H42N3 | 30.63±0.08 | 30.88±0.22 |
| H2A2N3 | 27.06±0.20 | 26.99±0.06 |
| H2B2N3 | 39.80±0.13 | 39.52±0.14 |

**Table S2**: End-to-end distance of closed structures (*d*_13_<10nm) and open structures (*d*_13_>10nm) for all histone tails with acetylation.

| **Tail Name** | **End-to-End Distance (d13<10nm)** | **End-to-End Distance (d13>10nm)** |
| --- | --- | --- |
| H31N1 | 39.04±0.31 | 38.87±0.18 |
| H41N1 | 36.19±0.09 | 36.25±0.06 |
| H2A1N1 | 25.12±0.05 | 25.12±0.02 |
| H2B1N1 | 37.58±0.08 | 37.71±0.04 |
| H32N1 | 40.14±0.36 | 40.13±0.08 |
| H42N1 | 36.31±0.20 | 36.36±0.04 |
| H2A2N1 | 25.30±0.06 | 25.15±0.03 |
| H2B2N1 | 37.84±0.12 | 38.25±0.04 |
| H31N2 | 39.09±0.39 | 39.58±0.11 |
| H41N2 | 36.34±0.07 | 36.20±0.04 |
| H2A1N2 | 24.98±0.04 | 25.14±0.02 |
| H2B1N2 | 37.76±0.09 | 37.81±0.05 |
| H32N2 | 39.74±0.25 | 39.95±0.16 |
| H42N2 | 36.05±0.04 | 36.34±0.06 |
| H2A2N2 | 25.06±0.06 | 25.16±0.03 |
| H2B2N2 | 38.20±0.11 | 38.18±0.03 |
| H31N3 | 39.44±0.14 | 39.48±0.11 |
| H41N3 | 36.25±0.10 | 36.38±0.14 |
| H2A1N3 | 25.27±0.04 | 25.11±0.02 |
| H2B1N3 | 37.84±0.09 | 37.74±0.04 |
| H32N3 | 39.78±0.38 | 39.15±0.18 |
| H42N3 | 29.92±0.06 | 30.18±0.05 |
| H2A2N3 | 26.66±0.07 | 26.66±0.04 |
| H2B2N3 | 40.80±0.16 | 40.58±0.04 |
